# Alkaline Phosphatase: An Old Friend as Treatment Target for Cardiovascular and Mineral Bone Disorders in Chronic Kidney Disease

**DOI:** 10.3390/nu14102124

**Published:** 2022-05-19

**Authors:** Mathias Haarhaus, Giuseppe Cianciolo, Simona Barbuto, Gaetano La Manna, Lorenzo Gasperoni, Giovanni Tripepi, Mario Plebani, Maria Fusaro, Per Magnusson

**Affiliations:** 1 Division of Renal Medicine, Department of Clinical Science, Intervention and Technology, Karolinska Institutet, Karolinska University Hospital, SE-14186 Stockholm, Sweden; mathias.loberg-haarhaus@regionstockholm.se; 2 Diaverum Sweden AB, SE-21537 Malmö, Sweden; 3 Nephrology, Dialysis and Renal Transplant Unit, IRCCS-Azienda Ospedaliero-Universitaria di Bologna, Alma Mater Studiorum University of Bologna, 40126 Bologna, Italy; giuseppe.cianciolo@aosp.bo.it (G.C.); simona.barbuto@studio.unibo.it (S.B.); gaetano.lamanna@unibo.it (G.L.M.); 4 Nephrology and Dialysis Unit, AUSL Romagna Infermi Hospital, 47923 Rimini, Italy; lorenzo.gasperoni3@gmail.com; 5 CNR-IFC, Clinical Epidemiology of Renal Diseases and Hypertension, Ospedali Riuniti, 89124 Reggio Calabria, Italy; gtripepi@ifc.cnr.it; 6 Laboratory Medicine Unit, Department of Medicine, University of Padova, 35128 Padova, Italy; mario.plebani@unipd.it; 7 National Research Council (CNR), Institute of Clinical Physiology (IFC), Via G. Moruzzi 1, 56124 Pisa, Italy; 8 Department of Medicine, University of Padova, Via Giustiniani 2, 35128 Padova, Italy; 9 Department of Clinical Chemistry, and Department of Biomedical and Clinical Sciences, Linköping University, SE-58185 Linköping, Sweden

**Keywords:** bone alkaline phosphatase, bone fractures, bone turnover, cardiovascular disease, end-stage renal disease, fracture risk, intestinal alkaline phosphatase, vascular calcification, vitamin K, vitamin K-dependent proteins

## Abstract

Alkaline phosphatase (ALP) is an evolutionary conserved enzyme and widely used biomarker in clinical practice. Tissue-nonspecific alkaline phosphatase (TNALP) is one of four human isozymes that are expressed as distinct TNALP isoforms after posttranslational modifications, mainly in bone, liver, and kidney tissues. Beyond the well-known effects on bone mineralization, the bone ALP (BALP) isoforms (B/I, B1, B1x, and B2) are also involved in the pathogenesis of ectopic calcification. This narrative review summarizes the recent clinical investigations and mechanisms that link ALP and BALP to inflammation, metabolic syndrome, vascular calcification, endothelial dysfunction, fibrosis, cardiovascular disease, and mortality. The association between ALP, vitamin K, bone metabolism, and fracture risk in patients with chronic kidney disease (CKD) is also discussed. Recent advances in different pharmacological strategies are highlighted, with the potential to modulate the expression of ALP directly and indirectly in CKD–mineral and bone disorder (CKD-MBD), e.g., epigenetic modulation, phosphate binders, calcimimetics, vitamin D, and other anti-fracture treatments. We conclude that the significant evidence for ALP as a pathogenic factor and risk marker in CKD-MBD supports the inclusion of concrete treatment targets for ALP in clinical guidelines. While a target value below 120 U/L is associated with improved survival, further experimental and clinical research should explore interventional strategies with optimal risk–benefit profiles. The future holds great promise for novel drug therapies modulating ALP.

## 1. Biology and Structure of ALP Isozymes and Isoforms

**Figure 1:**
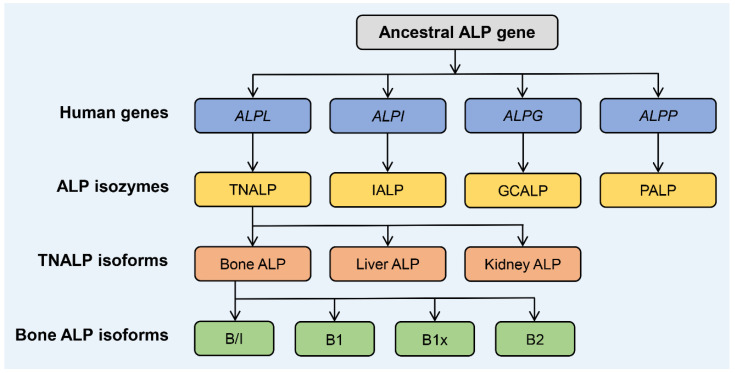
Four ALP isozymes are expressed in humans: tissue-nonspecific ALP (TNALP), intestinal ALP (IALP), germ cell ALP (GCALP), and placental ALP (PALP). The highest levels of human TNALP isoforms are expressed in bone, liver, and kidney tissues. Four BALP isoforms (B/I, B1x, B1, and B2) can be detected and quantified by weak anion-exchange high-performance liquid chromatography. The three BALP isoforms B/I, B1, and B2 are all detected in serum from healthy individuals. It should be noted that the B/I (bone/intestinal) ALP isoform is not a pure BALP isoform because it co-elutes with circulating IALP (approximately 70% BALP and 30% IALP) and comprises only approximately 6% of the total serum BALP activity. The fourth BALP isoform, B1x, is present in extracts of human bone tissue and exclusively in the serum of patients with different stages of CKD.

Alkaline phosphatase (ALP; EC 3.1.3.1) is a highly conserved enzyme that catalyzes the hydrolysis of phosphomonoesters, e.g., the endogenous substrate inorganic pyrophosphate (PPi), with an optimum activity at alkaline pH values. Four ALP isozymes are expressed in humans: tissue-nonspecific ALP (TNALP, a.k.a. liver/bone/kidney ALP), intestinal ALP (IALP), placental ALP (PALP, a.k.a. PLAP), and germ cell ALP (GCALP, a.k.a. placental-like ALP). The tissue-specific ALPs (i.e., IALP, PALP, and GCALP) are located on chromosome 2, bands q34.2–q37, with 90–98% sequence identity (Figure 1) [1]. The TNALP gene *ALPL* is located on the short arm of chromosome 1, band 1p36.12, with approximately 50% homology to the three tissue-specific ALPs, and it is at least five times larger, mainly due to intron size differences [2,3,4]. As the isozyme name “tissue-nonspecific” implies, TNALP is expressed ubiquitously and modified by post-translational glycosylation processes, to become isoforms that provide significant proteomic diversity and specificity relating to various tissues and cells. The highest levels of human TNALP isoforms are expressed in bone, liver, and kidney tissues [1]; with neutrophil granulocytes [5], brain [6], and vascular cells [7] as secondary sources of TNALP activity.

All ALP isozymes function as homodimers and are attached to the outer cell membrane (i.e., ALP is an ectozyme) by a glycosylphosphatidylinositol (GPI) anchor. The release of soluble (anchor-free) ALP to the circulation is achieved through cleavage by GPI-specific phospholipase D [8,9,10]. Several GPI–protein families are known but circulating GPI-specific phospholipase D is not active towards GPI-anchored proteins on the surface of intact cells [11,12]. The mechanistic basis of the targeting and subsequent activation to cleave GPI-linked proteins remains elusive, although some inhibitory elements and activators have been reported [13].

The homodimer of ALP has two active site regions and each site is dependent upon a metal triplet comprising one Mg^2+^ and two Zn^2+^ ions. Zinc can bind to all three sites, but binds particularly strongly to two of these sites, while magnesium occupies the other site per ALP monomer [14,15]. No ALP has been shown to be enzymatically active in a metal-free state. A fourth metal-binding site, occupied by one Ca^2+^ ion and present in all human ALP isozymes, is another structural feature of importance. Although distant from the active site, several clinically severe mutations in TNALP occur close to this calcium-binding site [16,17]. The functional implication of this non-catalytic calcium-binding remains, however, to be elucidated.

In serum, the bone ALP (BALP) and liver ALP isoforms are the most abundant TNALP isoforms, in approximately a 1:1 ratio, comprising more than 90% of the total ALP activity [18]. The remaining circulating ALP activity, 1–10%, is attributed mostly to IALP [19]. Several different analytical methods for separation and quantification of serum ALP isozymes and TNALP isoforms have been described over the years [20]. In particular, the development of commercial immunoassays for serum BALP has improved the usefulness and availability for clinical routine and research. Although a positive advance, users should be aware of that these immunoassays do have some weaknesses regarding standardization, inter-method variability, and cross-reactivity with liver ALP epitopes [20,21,22].

Four different BALP isoforms (B/I, B1x, B1, and B2) (Figure 1) can be separated and quantified by weak anion-exchange high-performance liquid chromatography [20,23]. Current immunoassays for determination of serum BALP cannot distinguish between the different BALP isoforms. It has been reported that the monoclonal antibodies used in these immunoassays have different affinities for the two major BALP isoforms B1 and B2 [21]. In addition, the used calibrators have significantly different proportions of the BALP isoforms when compared with each other, which increases the inter-method variability [24,25]. All four BALP isoforms are expressed in human bone tissue [10,26] and in vascular smooth muscle cells (VSMC); that is, cells that play a central role in the ectopic vascular calcification associated with increased activities of serum BALP [7,27]. The three BALP isoforms B/I, B1, and B2 are all detected in the circulation, with B1 and B2 representing the highest activities [18,28]. The B/I (bone/intestinal) isoform fraction is not a pure BALP isoform because it co-elutes with the IALP isozyme found in the circulation, comprising approximately 70% BALP and 30% IALP [10,29]. The B/I fraction represents only approximately 6% of the total BALP activity found in serum and is seldom of clinical interest, although exceptionally rare cases have been reported [30]. The fourth BALP isoform, B1x, is less common in the circulation and has, thus far, only been detected in serum from patients with chronic kidney disease (CKD) [31,32,33], with the highest activities in patients on dialysis treatment [34].

## 2. Function of BALP

**Figure 2:**
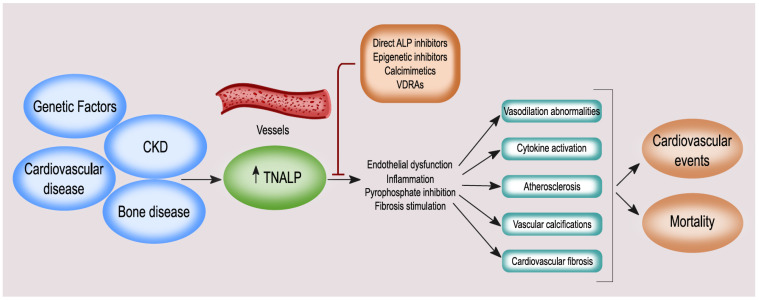
Roles of ALP in bone and cardiovascular disease.

BALP has a key role for the propagation of tissue mineralization and is expressed in osteoblasts, chondrocytes, and other mineralizing cell types such as calcifying VSMCs [23,35] (Figure 2). Loss of function mutations (mostly missense) in the TNALP gene *ALPL*, expressing BALP, cause hypophosphatasia (HPP; OMIM#: 146300, 241500, 241510), which is a rare inborn-error-of-metabolism [36]. HPP is characterized by defective bone and tooth mineralization, resembling rickets in children or osteomalacia in adults, and impaired vitamin B6 metabolism with increased risk of seizures and an accumulation of PPi, causing pseudogout and arthropathy. Low serum total ALP and BALP activities are pathognomonic findings. The clinical expression of HPP is highly variable and is classified into seven different forms: perinatal lethal, perinatal benign, infantile, childhood, adult, and odontohypophosphatasia. The prognosis for each of these HPP forms depends upon the severity of the skeletal disease, which reflects the age at presentation; i.e., the earlier a patient manifests skeletal symptoms, the more severe the HPP. The more severe forms (perinatal and infantile) are inherited as an autosomal recessive trait, while both autosomal recessive and autosomal dominant transmission can be found in the milder forms [37,38].

One of the primary roles for BALP during mineralization is to hydrolyze the mineralization inhibitor PPi into two phosphate molecules; thus, fine-tuning the local concentrations of PPi and organic phosphate to facilitate optimal local conditions for mineral precipitation and growth [35,39]. Too high concentrations of PPi results in significant hypomineralization of bone and teeth, with the clinical features of, for example, HPP and rickets [38]. Too low concentrations of PPi result in a pro-calcific environment, which promotes ectopic calcification in the vascular wall, causing vascular calcification [23]. The functional diversity of BALP is further augmented through the various isoforms of BALP, which are structurally and enzymatically different due to post-translational glycosylation modifications [40,41]. Besides inactivating PPi, BALP can also inactivate and regulate the calcification inhibitor osteopontin through dephosphorylation [42,43,44].

## 3. The Role of ALP/BALP in Inflammation, Metabolic Syndrome, and Proteinuria

ALP plays an important role in inflammation and metabolic syndrome [33]. Concerning the regulation of the immune and inflammatory response, TNALP is found in neutrophils, macrophages, and some lymphocytes. It has been demonstrated that interleukin (IL)-1β (in vitro) and bacterial endotoxins (in vivo and in vitro) increase the circulating ALP activity, producing a protective effect against bacterial cytotoxins [45]. While inflammation triggers circulating ALP, some inflammatory mediators have an inhibitory effect on BALP and mineralization. In an in vitro study aimed at evaluating the effect of C-reactive protein (CRP) on bone cells, Cho et al. demonstrated that CRP could suppress ALP and bone mineralization [46]. The concerted actions of tumor necrosis factor (TNF) α and IL-1β inhibit ALP and bone matrix mineralization in osteoblasts [47].

ALP can detoxify bacterial toxins and pro-inflammatory mediators, e.g., adenosine-tri-phosphate, through the dephosphorylation of various compounds. In some animal models of sepsis, ALP has been shown to improve survival rates by reducing inflammation and organ dysfunction [48,49,50,51]. Clinical studies of different intravenous ALP compounds in sepsis have produced promising renoprotective results. Heemskerk et al. carried out a phase IIa study on 36 patients admitted to intensive care with sepsis, by administering an intravenous bolus of bovine IALP or placebo. Patients treated with ALP had improved kidney function, associated with a lower risk of tubular damage. The authors hypothesized that the infusion of ALP inhibits the upregulation of inducible NO synthase (iNOS) and reduces production of the NO metabolite and renal enzymuria [52]. A subsequent study confirmed this result by demonstrating that, at 28 days, post-treatment creatinine clearance was significantly higher in patients treated with ALP. These patients also had a reduction in systemic inflammation markers such as CRP, IL-6, lipopolysaccharide binding protein and in the urinary excretion of renal damage markers such as kidney injury molecule-1 and IL-18 [53]. Conversely, the STOP-AKI trial, carried out on 301 patients admitted to intensive care for sepsis, showed that recombinant human chimeric IALP/PALP therapy does not have a significant effect on short-term renal improvement. However, this therapy significantly reduced the risk of major adverse kidney events at 60 days (27.0% vs. 39.7% in the placebo group; hazard ratio 1.8; *p* = 0.045) and 90 days (26.1% vs. 39.7% in the placebo group; HR, 1.9; *p* = 0.03) [51]. Further studies are needed to evaluate the impact of ALP on systemic and renal inflammation.

**Figure 3:**
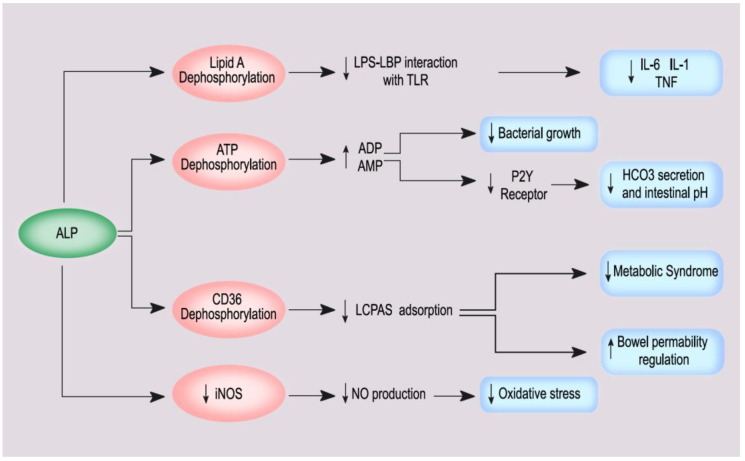
The main pathways of ALP on systemic inflammation, bowel and kidney.

In this context, it is important to analyze the role of IALP, which is involved in regulation of the intestinal microbiome, the inflammatory response, and the development of metabolic syndrome. IALP, located in the small intestine brush border, prevents the translocation of bacteria from the intestinal lumen to the mesenteric lymph nodes. A study in IALP knockout mice showed that they had less bacteria in their stool in comparison with wild-type mice [50]. Subsequent oral supplementation with IALP promoted colonization by commensal bacteria and inhibited the growth of pathogenic bacteria. Knockout mice for IALP also had an increased risk of obesity, dyslipidemia, and insulin resistance, which can promote metabolic syndrome [50]. Kaliannan et al. showed that IALP knockout mice had a greater risk of developing metabolic syndrome; moreover, the increase of IALP (endogenous or supplementing it orally) can prevent and reverse metabolic syndrome. Furthermore, in this study, IALP knockout mice suffered from type 2 diabetes [54] (Figure 3). In a case-control study, Malo et al. [55] compared IALP levels in the stools of 202 patients with type 2 diabetes and 445 healthy controls. Patients with type 2 diabetes had significantly lower IALP levels in their stool (approximately 48%) and obese patients with high IALP values did not develop diabetes. These findings suggest that the action of this enzyme could be protective against type 2 diabetes, and that a 50% loss of IALP activity could be predictive for the development of the disease [55].

Bulum et al. investigated patients with type 1 diabetes and found that serum ALP was associated with glomerular hyperfiltration, proteinuria and progression of CKD in the early stages of diabetic nephropathy [56]. Similarly, in a retrospective study on 299 patients with histologically diagnosed diabetic nephropathy, Zhao et al. analyzed the relationship between circulating ALP and renal outcomes, demonstrating that ALP is negatively associated with eGFR and positively associated with proteinuria [57]. In fact, patients who had ALP values greater than 97 U/L showed a 138% greater risk of developing end-stage renal disease (ESRD) or a 50% reduction in eGFR. In addition, a parallel increase was observed between the values of ALP and proteinuria. Patients with nephrotic syndrome had a poor outcome, i.e., 72% experienced a decrease in renal function. Circulating ALP was, therefore, shown to be independently associated with worse renal outcome. Finally, the levels of interstitial fibrosis and tubular degeneration were worse in patients with elevated ALP. A mechanism based on the sFRP2-Wnt signaling pathway was assumed to underlie the fibrosis [57]. It has also been speculated that the change in urinary ALP isozyme patterns may be associated with kidney disease [58]. In the kidneys, ALP is expressed on the brush border membranes of proximal tubular cells [59]. Since hyperfiltration and damaged tubular cells can increase urinary ALP levels, it has been speculated that this feature may be indicative of kidney disease. ALP is also expressed, in physiological conditions, on the endothelial, mesangial, and epithelial cells of Bowman’s capsule, and this could also increase the urinary excretion of ALP in hyperfiltration [23].

Increased circulating ALP is associated with cognitive impairment [60,61,62,63]. The underlying mechanisms may include a disturbance of vitamin B6 metabolism, since pyridoxal 5′-phosphate, the active form of vitamin B6, is an endogenous substrate for ALP and important for neuronal function in the brain [64]. In addition, ALP is also involved in cerebral neurotransmission [65] and contributes to the integrity of the blood–brain barrier [6,66]. While there may be a physiologic role for increased ALP in acute inflammatory states, e.g., sepsis, where increased ALP is associated with improved short-term outcome [67,68], elevated ALP in chronic disease is invariably associated with negative outcomes.

## 4. ALP and BALP: Vascular Calcification, Endothelial Dysfunction, Cardiovascular Disease (CVD), and Mortality

It is now acknowledged that vascular calcification derives from the pathological imbalance of pro and anti-calcific factors. The main pathways involved are the dysregulation of calcium-phosphate metabolism, inflammation, osteogenic gene expression, and transdifferentiation of VSMCs. In this mechanism, a central role is played by PPi, a powerful inhibitor of mineralization, regulated by the hydrolytic activity of ALP [69]. VSMCs play a primary role in vascular calcification, by transdifferentiation into an osteoblast-like phenotype that can be triggered by phosphate and calcium, mainly in presence of CKD, and derangement in mineral metabolism [7]. Calcified VSMCs show an increase in the activity of ALP and in the expression of the sodium-dependent phosphate co-transporter PiT-1, which allows phosphate to enter these cells [70]. The involvement of ALP or, more specifically, BALP isoforms in the calcification of soft/vascular tissues has been demonstrated in several experimental studies (Table 1) [7,69,71]. The role of ALP in vascular calcifications should be considered mainly in patients with CKD who suffer from alterations in bone metabolism; indeed, some studies have shown that the ALP values in these patients are very high [72,73]. In a randomized study of 137 hemodialysis patients, Shantouf et al. demonstrated that ALP was the only biochemical marker with a significant association with coronary artery calcification: ALP >120 U/L was associated with a high risk of coronary calcifications [74].

In addition to VSMCs, high ALP activities have also been described in endothelial cells [75]. It has been speculated that endothelial ALP may have an anti-inflammatory role, and associative studies suggested it may be involved in endothelial barrier dysfunction, arterial stiffness, proteinuria, or CKD progression [23]. Mice with endothelial ALP overexpression have been shown to have an increased risk of developing arterial hypertension and compensatory left ventricular hypertrophy [76] (Table 1). This finding was confirmed by a retrospective study of 12,539 patients without CVD, in which it was observed that elevated ALP levels were independently associated with arterial stiffness and an increased 10-year cardiovascular risk [77]. Similarly, Perticone et al. demonstrated in a study of 500 hypertensive patients without CKD, that elevated ALP values were associated with an alteration in endothelium-dependent vasodilation, an important measure of endothelial dysfunction [78].

Moreover, several circulating cell subsets have been identified; cumulatively named circulating calcifying cells, featuring an osteogenic phenotype defined by BALP and osteocalcin expression and sharing a common origin from bone-marrow progenitor. The circulating calcifying cells include circulating (mesenchymal) osteoprogenitor cells, circulating calcifying endothelial progenitor cells, and myeloid calcifying cells, and their presence has been demonstrated in diabetes, atherosclerosis, and CKD, where they could be involved in intimal calcification [79,82].

Fibrosis is a common pathological process in organ injury and failure, and has been estimated to cause at least one third of disease-related mortality worldwide [83]. In recent years, ALP has been suggested as a targetable promotor of organ fibrosis. In addition to its role in kidney fibrosis, ALP is involved in cardiovascular fibrosis. Cardiac TNALP is highly upregulated after a myocardial infarction and is associated with increased myocardial fibrosis [84]. Among the possible mechanisms linking TNALP to myocardial fibrosis, a stimulatory effect on the TGF-β/SMAD and ERK1/2 pathways has been suggested [85]. Upstream, an activation of Wnt-signaling is involved in the induction of ALP in cardiac fibroblasts [86,87]. Inhibition of ALP can effectively attenuate the myocardial fibrosis induced by myocardial infarction, implicating ALP as a possible therapeutic target for the prevention of heart failure after myocardial infraction [80].

The fundamental role of ALP in vascular calcification, endothelial dysfunction, and myocardial fibrosis identifies ALP as a promotor and possible treatment target for prevention of CVD. Kunutsor et al. evaluated 6974 participants (aged 28 to 75 years, with no known CVD) prospectively for 10.5 years; a non-linear association was found between serum ALP and CVD risk; partially dependent on CRP [88]. Similar results were found in another prospective study involving 3381 elderly people (aged 60–79 years) with no previous cardiovascular events. After an 11-year follow-up, higher ALP values were found to be associated with increased risk of coronary heart disease (adjusted hazard ratio for SD, 1.15 (1.03, 1.28); *p* = 0.01) and CVD events (adjusted hazard ratio for SD, 1.09 (1.01, 1.18); *p* = 0.02), as well as non-CVD mortality, even after adjustment for risk factors, including inflammation [89]. In addition, a prospective study of 1636 patients showed that patients with elevated ALP values had an increased risk of all-cause mortality and myocardial infarction, and a tendency for intrastent stenosis. It has been assumed that this may be related to the increased risk of coronary calcification, associated with high values of ALP, which can interfere with the re-endothelialization process, but this mechanism is not yet clear [90].

In contrast to the other ALP isozymes, IALP may be protective against CVD, in addition to its protective effect on metabolic syndrome and diabetes discussed above. In fact, a case-control study on 623 patients showed that ischemic heart disease was associated with lower fecal IALP activity [91]. This could be due to the increase in chronic systemic inflammation, with subsequent endothelial damage, which has previously been observed in studies on IALP-deficient mice.

Besides ALP, circulating BALP is also associated with vascular calcification and CVD, both in osteoporosis [92] and in CKD. A prospective cohort study of 135 patients with CKD (stages 1–5) demonstrated, after a 4-year follow-up, that cardiovascular events increased as CKD progressed and were associated with elevated circulating BALP levels. Interestingly, the risk of cardiovascular events was not associated with parathyroid hormone (PTH) values [93]. BALP is contained in vesicles shed by VSMCs and has a pro-calcifying effect, by promoting the deposition of hydroxyapatite crystals in the extracellular matrix. Vascular calcification causes CVD through various mechanisms, including vascular stiffening, and aortic and coronary calcifications [23]. Recently, the impact of BALP inhibition on vascular calcification was evaluated in a mouse model with CKD–mineral and bone disorder (CKD-MBD) [81] (Table 1). Furthermore, Barreto et al., in a prospective study of 64 hemodialysis patients, found that the progression of coronary calcifications was associated with BALP levels and low bone turnover (assessed by bone biopsy) [94]. Yan et al. also evaluated the relationship between BALP and aortic vascular calcifications in 156 hemodialysis patients and found that BALP was positively associated with aortic calcification and CRP [95].

Another intriguing topic is the presence of the new BALP isoform (B1x) in bone and exclusively in serum from CKD patients, and not in serum from healthy controls or patients with other metabolic bone diseases [20]. Some studies have shown an association between B1x, serum phosphate, and calcium phosphorus product, but not with eGFR and vitamin D status [34]. B1x is associated with low levels of ALP and the different BALP isoforms (B/I, B1, and B2), as well as low turnover in hemodialysis patients. Haarhaus et al. [96], in a study of patients in hemodialysis, evaluated the association between B1x, PTH, abdominal aortic calcification, and vascular stiffness for 2 years. They demonstrated how B1x was associated at baseline with vascular stiffness and time-varying improvement in vascular stiffness during a 2-year follow-up, but not with baseline, or change in, aortic calcification. Furthermore, B1x was associated with lower values of PTH and circulating ALP, but with better survival at 5 years, suggesting that B1x could be considered an indicator of low cardiovascular risk among patients with low bone turnover [96].

The association of ALP and mortality is another important topic to investigate. This was explored in some studies that found elevated ALP levels increased the risk of all-cause mortality, both when associated with an increase in CRP protein and independently of CRP [23]. High levels of ALP are associated with various conditions that can increase mortality, such as inflammation, vascular calcification, and CVD. The association between high levels of ALP, hospitalization, and all causes of mortality was studied in hemodialysis patients and in patients with earlier stages of CKD [33].

In 2011, Drechsler et al. [97] evaluated the impact of BALP on mortality in a study of 800 patients who had started dialysis. In this prospective study, elevated BALP levels were strongly associated with short-term mortality; indeed, patients in the highest tertile had a 5.7-fold increased mortality risk within 6 months. This applied to both cardiovascular mortality and all other causes. Elevated BALP levels were associated with increased mortality even in the long term (4 years). In this study the impact of ALP compared to BALP on mortality was also evaluated, it was shown that in the short term, elevated ALP levels were associated with increased mortality, but not in the long term. BALP may therefore have a higher impact on mortality than ALP in CKD [97]. However, this association may be specific for CKD, as BALP was not associated with mortality in the general population [98].

The impact of dialysis modality on the association of ALP with mortality was described in 99,323 patients in hemodialysis and 9244 in peritoneal dialysis (PD). PD patients had an increased mortality risk only when ALP was greater than 150 U/L. Compared to PD patients with normal ALP, the risk of death was higher for all ALP values in hemodialysis patient, especially when these were extremely high or low [99]. A recent prospective study of 1276 PD patients showed that the combination of high ALP and low PTH values is independently associated with increased cardiovascular and all-cause mortality [100]. Similar findings were found by Drechsler et al., who demonstrated that hemodialysis patients with high BALP and low PTH had a 2.8-fold increased risk of death within 6 months [97]. These findings may suggest that ALP is a better marker than PTH for predicting mortality and CVD. In fact, some studies have shown that ALP, and not PTH, was associated with a greater risk of coronary calcification in hemodialysis patients [94,101].

ALP is also associated with outcome after kidney transplantation. In kidney transplant recipients, pre-transplant ALP levels were predictive of post-transplant mortality; in fact, patients with high levels of ALP had a 64% risk of mortality and loss of the graft; however, this association could not be demonstrated for PTH [102].

Taken together, these findings suggest that it is useful to consider ALP or BALP as a reliable marker for bone metabolism, but also as a predictor of CVD and mortality in CKD patients, while ALP may be a better mortality predictor than BALP in individuals without CKD.

## 5. The Association between ALP, Bone Turnover, and Fracture Risk

Bone biopsy remains the gold standard for diagnosing bone turnover and the underlying pathology in patients with CKD, since histomorphometric analysis provides information on mineralization and volume [103,104]. Nonetheless, the procedure is not frequently performed, although it is only minimally invasive and complications are rare. The main issues that limit the wider use of bone biopsy are the availability of histomorphometrical expertise only at selected medical centers, time-consuming assessments, its ability to determine bone disease only at a single time point, and that there are no studies demonstrating links between bone biopsy and clinically relevant outcomes, e.g., fracture risk or mortality [105,106]. The ability of circulating bone markers to replace bone biopsies in clinical settings has repeatedly been examined. Markers of bone formation (osteoblast function) include BALP, osteocalcin, and PINP, while bone resorption markers (osteoclast function) include tartrate-resistant acid phosphatase isoform 5b (TRACP5b) and CTX [107]. To avoid the bias related to renal retention, markers of bone turnover that are not cleared by the kidneys should be considered in patients with CKD, i.e., BALP, intact (trimeric) PINP, and TRACP5b [108].

Coen et al. reported that BALP < 12.9 ng/mL had a sensitivity of 100%, a specificity of 94% and a positive predictive value of 72% in the prediction of low-turnover bone disease [109]. In the BONAFIDE study, carried out in hemodialysis patients who were treated with calcimimetics, the inclusion criteria of PTH ≥ 300 pg/mL, BALP > 20.9 ng/mL, and calcium > 8.4 mg/dL successfully excluded patients with adynamic bone, and most subjects had either mild or severe hyperparathyroid bone disease at baseline [110]. The association of ALP or BALP with bone outcomes has repeatedly been evaluated, both independently and in association with PTH [22]. Although levels of PTH and BALP tend to be lower in low-turnover bone disease and higher in high-turnover bone disease, BALP is considered to better reflect bone turnover; in particular, the bone formation rate. Diagnostic accuracy for both high- and low-turnover bone disease was higher for BALP than PTH, although there is some agreement that the bone turnover assessment can be refined by combining these molecules [109,111,112,113]. Disagreements between serum PTH and BALP levels are uncommon and may be related to variability in the measurements of BALP and PTH. Moreover, increased serum PTH levels with low BALP may reflect different degrees of the multifactorial skeletal resistance to PTH, just as the presence of aluminum overload, osteomalacia, Paget’s disease, or lytic bone lesions should be excluded [33,114,115].

B1x, one of the four BALP isoforms, circulates exclusively in serum of CKD patients and not in normal subjects [32,34]. A cross-sectional study carried out on 40 hemodialysis patients examined the relationship between serum BALP isoforms, PTH, and histomorphometric parameters. B1x was the only biochemical parameter that inversely correlated with the histomorphometric parameters of osteoblastic number and activity. Receiver operator characteristics curves showed that B1x can be used for the diagnosis of low bone turnover, whereas BALP and PTH are useful for the diagnosis of non-low bone turnover [32].

The ability of PTH and BALP to predict incidence of fractures has been assessed in a limited number of studies. In a large cohort study including 185,277 hemodialysis patients, a higher serum total ALP was significantly associated with the incidence of hip fracture, mainly in patients with lower PTH levels; conversely, serum ALP was not an independent predictor of the incidence of hip fracture among the patients in the highest quartile of PTH [116]. Similar results were found in a prospective study carried out on 485 ESRD patients. Incident fracture was associated with PTH levels either <150 pg/mL or >300 pg/mL compared with 150 to 300 pg/mL. In this study, higher BALP levels were associated with fracture risk, not only in patients with higher PTH, but also in patients with lower PTH [117]. These findings are in agreement with those of Atsumi et al., who showed that low PTH and high ALP was a high-risk for spine fracture in male hemodialyzed patients [118].

Neither KDOQI, nor the 2017 update of the KDIGO CKD-MBD guidelines, indicate a target value for ALP [119,120]. With respect to the extensive evidence for ALP as a risk marker for bone-related and cardiovascular outcomes, as well as for mortality, we suggest that ALP should be introduced as risk marker and treatment target in international guidelines [108,117,121,122,123,124,125].

## 6. Vitamin K and BALP

A promising area of research is represented by the possible interactions between vitamin K metabolism and BALP. Vitamin K is the term used for a family of fat-soluble compounds, which have various origin and function, share a common 2-methyl-1,4-naphthoquinone ring, but differ in the (lipophilic) side chains linked at the 3-position. The three main forms are vitamin K1 or phylloquinone (PK), vitamin K2 or menaquinone (MKn), and vitamin K3 or menadione. These three forms can be distinguished by the 3-position: PK has a phytyl side chain; whereas MK is characterized by a variable number of connected isoprenoid units (MKn); and finally, the menadione has no side chain, it is a synthetic analogue [126].

Each vitamer has a different food origin; whilst PK is found in high concentrations in green leafy vegetables such as spinach, cauliflower, and cabbage; MKn are mainly derived from fermented food and intestinal bacteria. In western diets, MKn can be found in fermented foods such as butter, beef liver, curdled cheese, and egg yolk [127]. The highest source of vitamin K2 (particularly MK-7) is a Japanese food named natto, which is produced by fermenting soybeans with Bacillus Subtilis. Furthermore, MKn are also produced by the intestinal bacterial flora: MK-10 and MK-11 are synthesized by Bacteroides, MK-8 by Enterobacteria, MK-7 by Veillonella, and MK-6 by Eubacterium lentum. The only exception is MK-4, which is produced from PK through a side chain removal/addition mechanism in specific tissues (pancreas, testes, and vessel wall) [126].

Vitamin K acts as a coenzyme of gamma-glutamyl carboxylase, which catalyzes the carboxylation (and thereby activation) of vitamin K-dependent proteins. Gamma-glutamyl carboxylase catalyzes the posttranslational gamma-carboxylation of glutamic acid (Glu) residues, within vitamin K-dependent proteins to gamma-carboxylated glutamic acid (Gla) residues [128,129]. To date, 17 members of the Gla protein family, involved in various biological processes, have been identified. In particular, the Gla family includes some proteins that regulate bone and vascular mineralization: matrix Gla protein (MGP), osteocalcin, growth arrest-specific protein 6, Gla-rich protein; two proline-rich Gla proteins, two transmembrane Gla proteins, periostin, and periostin-like factor [128]. Since some Gla proteins are involved in bone metabolism and vascular health, it has been hypothesized that their reduced carboxylation may lead to both impaired bone metabolism and vascular calcification [129].

**Figure 4:**
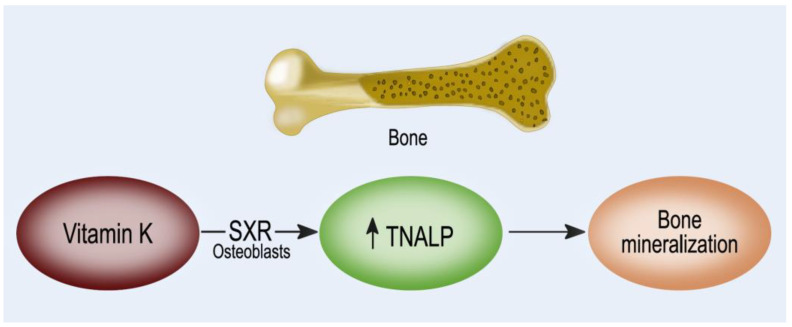
Vitamin K action as a ligand for the steroid and xenobiotic receptor (SXR).

The involvement of vitamin K in bone metabolism is not only linked to the gamma-carboxylation reaction. MK-4 binds to the nuclear receptor, steroid and xenobiotic receptor (SXR), and its murine ortholog, pregnane X receptor (PXR) (Figure 4). SXR is expressed in the liver, intestine, and human osteoblastic cells, and after binding to a ligand, SXR forms a complex with retinoid X receptor, which in turn binds to an SXR-responsive element on the target gene promoter that controls the transcription of osteoblastic markers: BALP, osteoprotegerin, osteopontin, and MGP [130,131]. Vitamin K1 is not able to activate SXR directly, suggesting that vitamin K1 may potentially contribute to this pathway only after being converted into MK-4.

MK-4, in an SXR-dependent manner, plays a pivotal role in bone metabolism by enhancing the expression of genes coding proteins such as matrilin-2, tsukushi, and CD14, which are involved in bone remodeling. Specifically, tsukushi encodes a protein that has a collagen-accumulating effect, while matrilin-2 is a widely distributed extracellular matrix protein-like collagen and CD14 regulates osteoblastogenesis and osteoclastogenesis by inducing differentiation of B cells [132,133]. The effect of vitamin K through SXR on bone collagen content may be important for bone quality. The material properties of bone, degree of mineralization, and microdamage accumulation are all influenced by collagen crosslinking formation. In particular, the type-I collagen fibers wired with crosslinks fibers form the framework that binds matrix proteins and mineral crystals. If the arrangement of collagen fibers is altered and the mineral crystal remains immature, these changes in material properties can cause an impairment of bone elasticity and strength [134,135]. Moreover, SXR/PXR promotes bone formation and blunts bone resorption, suggesting that SXR/PXR may play a pivotal role in maintaining bone homeostasis [133].

Recent evidence suggests that vitamin K regulates osteoblastogenesis and osteoclastogenesis through the nuclear factor κB (NF-κB) signal transduction pathway. NF-κB signaling plays a pivotal role in osteoclast development and resorption, and it also actively antagonizes osteoblast function and differentiation. Vitamin K2 enhances bone formation and suppresses bone resorption, by antagonizing basal and cytokine induced activation of NF-κB in a gamma-carboxylation-independent manner [136]. Furthermore, ALP activity was increased significantly in human Caco-2 cells by MK-4 treatment, disclosing similar biochemical properties to the typical IALP [137]. Several observational and interventional studies have reported that CKD patients undergoing conservative or artificial renal replacement therapy (hemodialysis or PD) suffer from subclinical vitamin K deficiency, related to dietary regimen and overall poor nutrient intake [138,139]. The effect of vitamin K on bone strength mainly manifests through the vitamin K-dependent carboxylation and consequent activation of osteocalcin and MGP. Osteocalcin is mainly produced, under the control of vitamin D, by osteoblasts and to a lesser degree by chondrocytes, and plays an essential role in the synthesis and regulation of the bone matrix [128,129,140]. MGP is considered one of the most effective endogenous inhibitors of vascular calcification in vivo and plays a multifaceted effect in bone turnover, since it not only promotes bone formation by upregulating Wnt/β-catenin signaling, but also inhibits osteoblast mineralization and affects bone mass by regulating the deposition of bone matrix [141,142].

Osteoblasts treated with vitamin K2, through a SXR activation mediated pathway, increase both ALP activity and the level of bone osteocalcin in the cell medium [130,143,144]. Higher ALP activity is associated with an improvement of both the organic matrix and mineral component of the bone, as well as the deposition of osteocalcin and hydroxyapatite in the bone. These studies suggest that SXR activators, by stimulating the synthesis of osteoblastic markers and deposition of bone may act as effective therapeutic agents that are able to improve bone quality and bone strength.

## 7. Treatment Strategies Targeting ALP

### 7.1. Modulation of ALP Expression: Serum ALP as Interventional Treatment Target

Different pharmacological strategies have the potential to modulate ALP. However, despite the strong evidence of ALP and BALP as risk factors for CVD, bone-related outcomes, and mortality, no specific treatment targets have been established in prospective interventional trials. Differences in the methodology used to detect ALP and BALP and differences in the normal ranges and reported units between assays may be possible reasons for this, albeit these obstacles can be overcome; as demonstrated by treatment goals for PTH in CKD, defined as multiples of upper limits of the normal values in different guidelines. An additional explanation may be that, although ALP and BALP have long been established as biomarkers, their biological role in different physiologic or pathophysiologic processes, e.g., tissue calcification, fibrosis, inflammation, and neurotransmission has only recently been explored. Thus, the relevance of the modulation of ALP and BALP towards specific activity levels in serum has yet to be determined.

Some indication of suitable target levels can be found in observational studies demonstrating a survival benefit for ALP levels < 120 U/L, in opposition to an incremental and linear relationship between higher total AP (>120 U/L) and all-cause death hazard ratio, which has been described in hemodialysis patients, contrary to the U-shaped curve describing the relationship between PTH and mortality [145,146,147].

Few studies have examined the association of changes of ALP over time with outcome. Soohoo et al. [148] demonstrated a trend towards better survival for a decrease of ALP by ≥30 U/L during 3 months, from low levels (≤80 U/L); whereas levels above 120 U/L were associated with increased mortality, independent of the direction of ALP change. This was contrasted by PTH, which demonstrated an increase in mortality risk for values that decreased from 150 pg/mL [148].

### 7.2. Effect of ALP Modulation on Clinical Outcomes: From CKD-MBD Treatment to Anti-Fracture Treatment

#### 7.2.1. Apabetalone

Some studies have recently demonstrated an association of decreasing serum ALP in apabetalone-treated patients, with improved cardiovascular outcome in patients with CVD [149] and improved kidney function in a subgroup of patients with CKD [150]. While these studies were not designed to target ALP, they may currently be the best available evidence for a beneficial effect of the pharmacological lowering of ALP on clinically relevant outcomes. The bromodomain and extraterminal (BET) family of proteins is characterized by the presence of two tandem bromodomains and an extraterminal domain [19]. A bromodomain is an approximately 110-amino-acid protein domain that recognizes acetylated lysine residues. Bromodomains are responsible for transducing the signal carried by acetylated lysine residues and translating it into various normal or abnormal phenotypes [151]. BET inhibitors are a class of epigenetic modulators that block the interaction of BET proteins with acetylated histones or transcription factors; thus, influencing the expression of target genes [152]. Apabetalone is an orally available BET inhibitor that hampers inflammation, vascular calcification, and fibrosis. Apabetalone has been shown to directly and indirectly inhibit ALP expression and activity in different tissues, including cardiovascular cell types [153,154]. While other BET inhibitors have the potential to improve bone mineral density (BMD) [19], the effect of Apabetalone on bone has not yet been studied.

#### 7.2.2. Vitamin D

Treatment with vitamin D can reduce ALP [155,156]. However, only one study has determined the effect of ALP modulation by vitamin D on clinical outcomes in CKD. Treatment with alphacalcidol improved BMD and reduced BALP in patients with pre-dialysis CKD [157].

#### 7.2.3. Phosphate Binders

In the BRiC study, treatment with sevelamer for 1 year increased ALP, compared to treatment with calcium acetate; while the progression of coronary calcification was similar in both groups [158]. The term attenuation, using electron beam CT, refers to the degree of absorption of X-ray beams passing through a body part, and can be considered a surrogate marker of BMD; therefore, a denser tissue will show a higher attenuation. In a post hoc analysis of a 52-week randomized trial carried out on 111 hemodialysis patients, thoracic vertebral trabecular bone attenuation decreased in patients treated with calcium-based phosphate binders, while it was unchanged in sevelamer treated patients [159]. After 1 year of treatment, ALP and BALP were significantly higher in sevelamer-treated patients than in calcium-treated patients. While the change of ALP and BALP under sevelamer treatment correlated positively with bone attenuation, ALP, but not BALP, correlated negatively with bone attenuation under calcium salt treatment. No trials with other phosphate binders have determined relationships between ALP or BALP modulation and clinically relevant outcomes.

#### 7.2.4. Calcimimetics

In a large post hoc analysis of phase III trials, a greater proportion of dialysis patients on cinacalcet for 1 year achieved a ≥20% reduction of ALP than controls [160]; however, the effect on other clinical outcomes was not determined. While a decline of ALP under cinacalcet treatment was also described by another study on hemodialysis patients, BMD remained unchanged [161]. Cinacalcet treatment induced an increase of BALP and a decrease of kidney function and PTH after 1 year of treatment in kidney transplant recipients; however, interrelationships between these factors were not determined [162]. Moreover, in primary hyperparathyroidism, treatment with cinacalcet induced a slight increase of BALP, while no changes of BMD were detected [163].

#### 7.2.5. Denosumab

The RANK-ligand inhibitor denosumab inactivates osteoclasts and through downregulation of osteoclast–osteoblast coupling factors also affects osteoblasts, resulting in reduced gene expression of ALP [164]. Denosumab suppressed BALP and other bone turnover markers for up to 8 years, while total hip and lumbar spine BMD increased continuously in postmenopausal women [165]. Although the follow-up times in CKD studies were considerably shorter, they demonstrated similar results of suppressed ALP or BALP and an improvement of BMD in dialysis patients, patients with CKD, and after kidney transplantation [166,167,168]. On the other hand, the effect of denosumab on PTH levels was variable in the general populations, as well as in CKD and kidney transplant recipients. Some studies recently reported a marked and persistent increase in PTH, independently of 25-hydroxyvitamin D levels [168,169,170]. While the increase in PTH can exert an osteoanabolic effect, acting exclusively on the osteoblasts, osteoclastic reabsorption is completely inhibited by denosumab. It is not clear whether the compensatory increase in PTH is beneficial for bone or not during denosumab treatment, and further research is needed to clarify this finding. Neither in postmenopausal women nor in dialysis patients was the suppression of bone turnover markers in general, and more specifically BALP or ALP by denosumab, associated with an increase in vascular calcification, arterial stiffness, or cardiovascular events [167,171].

#### 7.2.6. Bisphosphonates

Bisphosphonates have a high affinity for mineralized bone and suppress bone turnover by inhibiting osteoclast activity after internalization, during the process of bone resorption. Although a direct effect on osteoblasts and even on ALP was observed experimentally, the clinically observed inhibitory effect on bone formation may primarily be indirect through the suppressive effect on osteoclast activity [172]. In contrast to denosumab, inhibition of bone turnover is less severe, but can last for a long time, and even after discontinuation of treatment, since bisphosphonates remain active and accumulate in bone tissue for several years [173]. Studies in patients with CKD or ESRD on dialysis have demonstrated variable effects on circulating ALP activity and BMD, but a more consequent stimulating effect on PTH [174,175]. Although bone turnover is also suppressed in patients with advanced CKD, no unfavorable effect on vascular calcification or cardiovascular complications has been described [176,177]. In fact, a recent meta-analysis suggested a favorable effect of bisphosphonates on cardiovascular events in osteoporotic patients [178].

#### 7.2.7. Teriparatide

The bone anabolic PTH analogue teriparatide increases bone formation and BMD and demonstrates a consistent fracture risk reduction in osteoporosis. Teriparatide induces skeletal and circulating ALP and BALP activity. In pilot studies, the effect on BMD in CKD was impressive. However, there are uncertainties regarding dosing and treatment duration, as different dosing regimens were applied in CKD studies, none of which administered the full recommended dose for non-CKD patients with osteoporosis [179,180,181]. The clinical effect of teriparatide on vascular calcification has not been studied, and the sparse experimental evidence is inconclusive [182,183]. Two case reports of an induction of calcifylaxis in non-CKD patients may warrant caution of its use in CKD. Abolaparatide is a novel PTH analogue that improves BMD and reduces fracture risk in osteoporotic patients [184]. No evidence exists for its use in CKD or its effect on ALP or BALP.

#### 7.2.8. Romosozumab

The sclerostin inhibitor romosozumab blocks the Wnt inhibitor sclerostin and induces both a predominating anabolic and an antiresorptive effect in bone. It has been shown to improve BMD and reduce fracture risk in osteoporotic patients. Circulating sclerostin is increased early in CKD and exerts its effect in target organs, such as the skeleton and the vasculature, possibly contributing to a low-turnover bone phenotype [185]. The vascular effect of sclerostin in CKD is unclear, as it has been associated with the development of, as well as protection against, vascular calcification [186,187]. Given the anabolic effect in bone, a stimulatory effect on ALP activity by romosozumab can be speculated. However, no studies have specifically described the effect of romosozumab on ALP or BALP. Safety signals regarding cardiovascular events in some studies involving osteoporotic patients without CKD warrant precaution for its use in CKD [188]. A first observational study of 1 year of romosozumab treatment in prevalent Japanese dialysis patients demonstrated a significant increase of BMD, while the incidence of new cardiovascular events was nominally lower than in a sex- and age-matched control group [189]. Remarkably, 61.5% of romosozumab-treated patients were pre-treated with bisphosphonates, which was stopped at initiation of romosozumab treatment, and 37.5% were on calcimimetics treatment. Both ALP and PTH increased in the romosozumab-treated patients. Although reassuring, evidence from additional carefully-designed controlled studies is needed to increase the understanding of the potential advantages and risks of romosozumab treatment in advanced CKD [189].

## 8. Future Perspectives

Circulating ALP is an independent risk marker for CVD and mortality in the general population and in CKD. The ubiquitous expression of ALP and its involvement in several pathophysiologic processes associated with bone disease, CVD, and CKD progression renders ALP a suitable target for multifactorial approaches. Although the currently available biomarkers cannot reliably predict bone turnover or mineralization in most patients with CKD, ALP may play a role in prediction of fractures and in the monitoring of therapeutic responses. In particular, total ALP and BALP are suitable bone formation markers in CKD, since they are not affected by GFR and show a linear and direct relationship with bone and vascular outcomes in CKD [22,108,122]. Future studies should aim for a definition of the role of the different BALP isoforms in bone disease and vascular calcification, as well as identifying specific biomarker patterns as a function of bone turnover.

Considering that any chronic inflammatory state can be expected to affect bone cell activity by stimulating osteoclast maturation and activity, and increasing dickkopf-1 and sclerostin that in turn inhibit bone formation [82], the effects of mediators of inflammation on ALP and BALP isoform activity should also be evaluated. In particular, circulating B1x can actually indicate a state of low bone turnover without an increase of circulating ALP activity associated with improved survival. Another issue to speculate on is, whether the association of an increased activity of circulating ALP with mortality is mainly driven by systemic inflammation, a neutral or even positive bone balance, or direct effects of cardiovascular calcification on the circulating BALP isoforms. A possible modulatory role of ALP and BALP in fibrosis is an interesting new field of research, and the recent finding of an association of B1x with pulse wave velocity can point towards a possible clinical relevance [96]. To date, no studies have investigated the role of glycosylation differences among the BALP isoforms on the potential of ALP to modulate tissue fibrosis. Experimental studies with BET inhibitors and miRNAs suggest a wider therapeutic potential for epigenetic modulation of ALP [19]. Further research is required to definitively establish ALP as a clinical treatment target and to elucidate the effect of lowering serum ALP towards specific target levels on clinical outcomes. In particular, the development of molecules able to inhibit and modulate TNALP could be effective in the prevention of cardiovascular complications, while the systemic administration of IALP for the treatment of acute inflammatory disorders could be a promising approach, aimed at reducing the increased mortality associated with CKD.

Some studies are currently starting to evaluate the effect of ALP in many fields. The APPIRED-III study (phase III, randomized, quadruple-blind, placebo-controlled), involving 1250 participants, is evaluating the impact of the administration of bovine IALP on the incidence of acute kidney injury, in patients undergoing cardiopulmonary bypass. Secondary outcomes are the impact on systemic inflammation (evaluating IL-6, IL-8, IL-10, TNF-α as markers) and relative costs (NCT03050476). At the same time, the Revival study (phase III, double-blind, randomized, placebo controlled) is also evaluating the effect of recombinant ALP on 1600 intensive care patients with sepsis and acute kidney injury. The primary endpoint is to confirm the benefit on mortality, already seen in the STOP-AKI study and to demonstrate a reduction in all-cause mortality at 28 days (NCT04411472). In Japan, a study is underway (phase I, double-blind, randomized, monocentric) on 32 healthy subjects, to evaluate the pharmacokinetics, safety, and tolerability of the infusion of recombinant human ALP versus placebo (NCT04923282). Finally, a study (phase I, open-label), in 15 adult patients with HPP, is ongoing to evaluate the safety, tolerability, pharmacokinetics, pharmacodynamics, and immunogenicity of the drug ALXN1850 (enzyme replacement therapy) administered intravenously or subcutaneously (NCT04980248).

## 9. Conclusions

In conclusion, the current review identifies ALP as an important risk factor for cardiovascular and bone-related outcomes, as well as all-cause mortality. Mounting evidence has identified the involvement of ALP in underlying pathomechanisms, e.g., chronic inflammation, skeletal and ectopic mineralization, endothelial dysfunction, and tissue fibrosis. Recent experimental and clinical studies have demonstrated the positive effects of lowering ALP on relevant clinical outcomes. Circulating ALP and BALP are not influenced by residual renal function and are less variable than PTH and, thus, more suitable as biomarkers and treatment targets to guide the management of CKD-MBD. Established treatment strategies for the management of CKD-MBD can modulate circulating ALP. In addition, novel pharmacologic agents are under development that can inhibit ALP more directly. Novel clinical guidelines should, therefore, integrate specific treatment targets for ALP and/or BALP. In addition, we propose that future interventional studies in CKD-MBD with pharmacologic agents that can modulate ALP should always include specific treatment targets for ALP.

## Figures and Tables

**Table 1:** The role of ALP in vascular calcifications: experimental studies.

Study	Model	Results	Reference
“Calcifying human aortic smooth muscle cells express different BALP isoforms, including the novel B1x isoform”	Calcifying human aortic smooth muscle cells (HAoSMC) cultivated for 30 days in a medium containing 5 or 10 mmol/L of glycerophosphate in the presence or absence of the specific inhibitor of ALP (tetramisole).	All bone-specific ALP isoforms (B/I, B1x, B1 and B2) were identified in HAoSMC; calcification was associated with an increase in isoforms B/I, B1x and B2.	[7]
“Pathophysiological role of vascular smooth muscle ALP in medial artery calcification”	Mouse model with overexpression of human TNALP in vascular smooth muscle cells.	These mice had vascular calcifications, hypertension, cardiac hypertrophy and early mortality. Administration of ALP inhibitor led to an improvement in cardiovascular outcome and life expectancy.	[69]
“Impaired calcification around matrix vesicles of growth plate and bone in ALP-deficient mice”	Knockout mice for the ALP gene (which includes the transcription of BALP).	TNALP Knockout mice showed significant hypomineralization. TNALP is an important promoter of bone mineralization.	[71]
“Cellular localization of endothelial ALP reaction product and enzyme protein in the myocardium”	Myocardial tissue samples of different species (human, rat, and pig).	In the myocardium, ALP was localized in all the species studied, mainly in the plasma endothelial membrane and in the pinocytotic vesicles.	[75]
“Transgenic overexpression of TNALP in vascular endothelium results in generalized arterial calcification”	Mice with endothelial ALP overexpression.	Mice develops arterial calcifications, increased blood pressure, and compensatory left ventricular hypertrophy. This model demonstrates how ALP positive endothelial cells can also promote vascular calcification.	[76]
“Widespread increase in myeloid calcifying cells contributes to ectopic vascular calcification in type 2 diabetes”	Circulating procalcifying cells (osteocalcin and BALP positive) from 100 patients with or without diabetes and CVD.	There is a subpopulation of pro-calcifying cells that come from the myeloid lineage and retain monocyte/macrophages markers (myeloid calcifying cells). They are overrepresented in the blood of patients with type 2 diabetes and in atherosclerotic lesions.	[79]
“TNALP inhibition attenuates cardiac fibrosis induced by myocardial infarction through deactivating TGF- β1/Smads and activating p53 signaling pathways”	Sections of heart of patients and rats with myocardial infarction.	Inhibition of TNALP regulated cardiac fibrosis and exerted an antifibrotic effect through AMPK-TGF-β1/Smads and p53 signals.	[80]
“Inhibition of TNALP protects against medial arterial calcification and improves survival probability in the CKD-MBD mouse model”	CKD-MBD mouse model.	In mice with inhibited ALP, calcifications were blocked. Survival was 100%, compared to those not treated with the inhibitor (57% survival).	[81]

## Abbreviations

ALP	alkaline phosphatase
BALP	bone alkaline phosphatase
BET	bromodomain and extraterminal
BMD	bone mineral density
CKD	chronic kidney disease
CKD-MBD	chronic kidney disease-mineral bone disorder
CRP	C-reactive protein
CVD	cardiovascular disease
ESRD	end-stage renal disease
GCALP	germ cell alkaline phosphatase
GPI	glycosylphosphatidylinositol
HAoSMC	human aortic smooth muscle cells
HPP	hypophosphatasia
IALP	intestinal alkaline phosphatase
IL	interleukin
iNOS	inducible NO synthase
MGP	matrix Gla protein
MKn	menaquinone
NF-κB	nuclear factor κB
PALP	placental alkaline phosphatase
PD	peritoneal dialysis
PK	phylloquinone
PPi	inorganic pyrophosphate
PTH	parathyroid hormone
SXR	steroid xenobiotic receptor
TNALP	tissue-nonspecific alkaline phosphatase
TNF	tumor necrosis factor
TRACP5b	tartrate-resistant acid phosphatase isoform 5b
VSMC	vascular smooth muscle cell
